# Colonoscopic Findings in Peruvian Patients with Chronic Diarrhea

**DOI:** 10.1371/journal.pone.0046690

**Published:** 2012-10-18

**Authors:** Javier Villafuerte-Gálvez, María Isabel Sotelo-Olivera, Jaime Cok, Alejandro Piscoya-Rivera, Jorge Huerta-Mercado

**Affiliations:** 1 Medical School, Universidad Peruana Cayetano Heredia, Lima, Peru; 2 Sociedad Científica de Estudiantes de Medicina Cayetano Heredia (SOCEMCH), Lima, Peru; 3 Department of Pathology, Hospital Nacional Cayetano Heredia, Lima, Peru; 4 Department of Preclinical Science and Diagnostic Aid, Universidad Peruana Cayetano Heredia, Lima, Peru; 5 Gastroenteology Unit, Department of Medicine, Hospital Nacional Cayetano Heredia, Lima, Peru; 6 Department of Clinical Medicine, Universidad Peruana Cayetano Heredia, Lima, Peru; National Cancer Institute, United States of America

## Abstract

**Objective:**

To report the colonoscopic and pathological findings in patients with chronic diarrhea from a gastroenterology unit during approximately 3 years in a general teaching hospital located in Lima-Peru.

**Materials and Methods:**

Patients with chronic diarrhea as the motive for colonoscopy from March 2008 to December 2010 were selected from the colonoscopy report computerized database. Colonoscopic findings were registered. Biopsies taken during the procedure were prospectively reviewed.

**Results:**

226 patients were included, of which 162 (71.7%) had a colon biopsy available. The average age of the patients was 53.6±16.36. 85.8% of patients were reported to have a normal colon. 14.8% of patients were found to have a normal colonic mucosa or mucosal edema, 35.8% of patients had lymphocytic colitis and 28.4% had paucicelular colitis.

**Conclusions:**

The majority of colonoscopies were reported with unremarkable macroscopic findings. Lymphocytic colitis was unusually frequent compared to previous reports.

## Introduction

Chronic diarrhea afflicts around 7.3% of adults in the United States [1] and as much as 14.2% of the elderly [2], only limited data exists regarding the burden of disease in developing countries. The primary causes may vary depending on socio-economic factors, referral status as well as the practice setting. Chronic diarrhea in the developing world has been frequently attributed to chronic infections, especially parasitosis [3], [4]. Studies exploring the causes of chronic diarrhea in developing countries are scarce and might not be generalizable from one setting to another.

Besides the burden of their symptoms, patients with chronic diarrhea in the developing world often have to endure going through costly and invasive testing which may not finally warrant them a diagnosis with an effective treatment or with a treatment they may afford.

Recently, microscopic colitis was reported as a frequent finding in chronic diarrhea patients from a referral private practice institution in Peru [5]. The frequency reported exceeded largely the one reported in previous studies in the US, Chile and Mexico [6], [7], [8], [9], [10].

The subjects studied in the mentioned report likely differ significantly in their socio-economic status from the patients at our institution which is a teaching hospital situated in the underserved northern outskirts of Lima, Peru. Microscopic colitis is a group of conditions for which effective and safe treatments exists [6], [7]; unfortunately one of the main treatment options, budesonide, is currently not available in the country. Additionally, there have been recent reports of another entity named paucicellular lymphocytic colitis [11] which has not been yet been widely described in different geographical settings.

Assessing the magnitude of the phenomenon in a different and larger subset of patients in Peru can be the basis for further studies which may have an impact in local practice guidelines, market availability of the treatment and perhaps public policy.

Our objective was to study the colonic and terminal ileal macroscopic and mucosal histopathological findings in a group of patients undergoing colonoscopy as part of the workup for chronic diarrhea.

## Materials and Methods

### Study Design and Population

We conducted a retrospective case-series study at the Gastroenterology Unit of Hospital Nacional Cayetano Heredia in Lima, Peru. Through the unit's computerized colonoscopy database we included all patients 18 years old and above who underwent colonoscopy from March 1st 2008 to December 31st 2010 having chronic diarrhea as the sole indication for the procedure. We excluded patients documented to have an incomplete colonoscopy (failure to achieve distal ileum intubation) or who had a positive history for HIV infection.

The working definition used by the gastroenterology unit for chronic diarrhea was the presence of loose or frequent (≥3/day) bowel movements during four weeks or more that was a clear change from the patient's baseline. Patients with chronic diarrhea are regularly tested for stool ova and parasites before being referred to our unit for colonoscopy. Stool culture is not routinely performed in patients with chronic diarrhea in our setting due to its poor reliability, high cost and low diagnostic yield in this clinical situation.

### Data collection

By means of the same database we reviewed the patient's colonoscopy reports and recorded the following variables: age, sex, gross colonoscopic findings, findings on distal ileoscopy, presence of polyps or diverticula and whether a colonic or ileal mucosal biopsy was taken.

Based on a pilot study the gross colonoscopic findings where recorded as one of the following: elevated lesion, ulcerated/erosive lesion, mixed (elevated and ulcerated/erosive) lesion and normal colonoscopy (none of the lesions above mentioned). The findings in the distal ileum were recorded under the same categories but one specific category was added: abnormal vascular pattern.

When the colon was within normal limits, two biopsies were taken from ascending, transverse and descending colon respectively. When specific lesions (i.e. ulcerated, elevated or mixed) were found biopsies of only the specific lesions were taken according to the clinical expertise of the endoscopist.

The pathology slides corresponding to each patient's biopsies where anonymized and prospectively reviewed by a single experienced GI pathologist under standardized pathological criteria [11], [12]. Granulomatous colitis was defined as the presence of formed granulomata or the tendency to form granulomata whether or not mucosal ulceration was present. The histological diagnosis of lymphocytic colitis or paucicellular colitis was made when at least one of the included colonic samples met the standard criteria. If a patient's slideset was not found in the hospital's pathology slide repository we recorded that no biopsy was available.

### Data Analysis

A database was generated with the recorded data using OpenOffice Calc 3.2 for Windows. The data analysis was performed with SPSS v.17 for Windows. We determined the relative frequencies for both macroscopic and mucosal histopathologic findings as well as the mean and SD for age. A normal distribution of age data was confirmed before using the t-Student test to compare the means of ages in different subgroups. Chi-square and Fisher's exact test were used as necessary to explore possible associations between categorical variables.

### Oversight

This study was a retrospective review of previously existing procedure data and pathology slide repositories. Since data and slides had been collected prior to the beginning of the study informed consent was not necessary. The protocol of this study was reviewed and approved by Universidad Peruana Cayetano Heredia's Institutional Review Board. No patient identifying information was recorded in the database.

## Results

We included 226 patients in the study. 180 colonic mucosal biopsies were performed (79.65%). The colonic slidesets were available for 162 patients (70.8%) and were prospectively reviewed. The patient's mean age was 53.6±16.36 years. 51.8% of the patients were female.

We found that 85.8% of patients who underwent colonoscopy had a macroscopically normal colon (Table 1). Among the 32 patients that had an abnormal colonoscopy, 23 (71.9%) had an ulcerated/erosive lesion, 7 (21.9%) had mixed lesions and 2 (6.2%) had elevated lesions. There was no significant difference between the age of patients with a macroscopically normal and an abnormal colon (p = 0.067).

**Table 1:** Colonoscopic findings.

		Colon		
		Abnormal n	Normal n	Total n (%)
Distal ileum	Abnormal n	9	12	21 (9.3%)
	Normal n	23	182	205 (90.7%)
	Total n (%)	32 (14.2%)	194 (84.8%)	226 (100%)

A macroscopically normal distal ileum was found in 90.7% of the patients. Out of the 21 patients with an abnormal distal ileum 11 (52.4%) had an abnormal vascular pattern as the only finding, 6 (28.6%) had ulcerated/erosive lesions and 4(19.0%) has mixed lesions. (Table 2)

**Table 2:** Specific colonoscopic findings in patients with an abnormal colon.

	n	%
Erosive/Ulcerated Lesion	23	71.9
Elevated Lesion	7	21.9
Mixed Lesion	2	6.2
Total	32	100

There was a marginally significant association between being male and having a macroscopically abnormal colon (χ2 = 4.066, p = 0.044). Incidental findings not related to chronic diarrhea such as presence of colonic polyps in 10.2% of patients and presence of colonic diverticula in 10.2% of patients was recorded.

Concerning the colonic mucosal histopathological findings 14.8% of the 162 patients who had a biopsy available had a normal colonic mucosa, 35.8% had lymphocytic colitis, 28.4% had paucicellular lymphocytic colitis, 4.3% had a granulomatous colitis, 3.7% has a diffuse/focal active colitis, 3.1% has cytopathic changes suggestive of CMV colitis, 2.5% had neoplasia (colon adenocarcinoma, lymphoma, signet-ring cell carcinoma), 2.5% had ulcerative colitis and the remaining 4.9% had other findings (among them solitary rectal ulcer, colonic spirochetosis, colonic histoplasmosis, eosinophilic colitis, pseudomembranous colitis) (Table 3).

**Table 3:** Colonic histopathological findings.

	n	%
Normal	24	14.8
Lymphocytic Colitis	58	35.8
Paucicellular Lymphocytic Colitis	46	28.4
Granulomatous Colitis	7	4.3
Focal/Diffuse Active Colitis	6	3.7
Cytopathic changes suggestive of CMV Colitis	5	3.1
Neoplasia	4	2.5
Ulcerative Colitis	4	2.5
Other	8	4.9
Total	162	100

The mean age of patients with lymphocytic colitis (58.5±15.9 years) was significantly higher (p = 0.012) than the mean age of patients without that diagnosis (52.1±14.1 years). An age 50 years old or above was significantly associated with the finding of lymphocytic colitis (χ2 = 4.838, p = 0.028). Having a macroscopically normal colon was significantly associated with having a normal colonic mucosa (χ2 = 6.143, p = 0.013)as well as with the finding of lymphocytic colitis (χ2 = 5.295, p = 0.021). Having an abnormal colonoscopy was significantly associated with the findings of ulcerative colitis (Fisher's p = 0.001) as well as with granulomatous colitis (Fisher's, p = 0.002).

Regarding the histopathological findings in the terminal ileum, 11 of the 21 patients (52.4%) had a biopsy available. Out of the subjects with a biopsy available 7 (63.6%) had an abnormal vascular pattern as the only distal ileal finding. A normal terminal ileal mucosa was the most frequent finding with 8 (72.7%). Out of the 3 (27.3%) abnormal ileal mucosal biopsies, 2 corresponded to isolated intraepithelial lymphocytosis and one to isolated intestinal lymphangiectasia. (Table 4)

**Table 4:** Terminal ileal histopathological findings.

	n	%		
Normal	8	72.7		n
Abnormal	3	27.3	Intraepithelial lymphocytosis	2
			Intestinal Lymphangiectasia	1

## Discussion

One of the strengths of the study is being, to our knowledge, the largest case-series of patients with chronic diarrhea studied in Peru. However, as in most hospital based case series, due to referral bias, the study population may not entirely reflect the community. Patients suffering from chronic diarrhea who did not undergo colonoscopy are not represented in this study.

The histopathological findings were only available for 70.8% of the 226 patients who underwent colonoscopy. Neither our institution nor our country has specific guidelines for the study or treatment of patients suffering from chronic diarrhea. Whether the patients are referred to colonoscopy, have colonic mucosal biopsies taken or not depends on the treating physician's individual clinical criteria. Since the patient is responsible for the cost of processing the biopsy as well as the transportation to the pathology lab, often a small number of biopsies are lost or never processed. We acknowledge the potential bias that these circumstances may introduce in the conformation of our group of subjects.

The mean ages and gender proportions between our study and prior ones did not differ notably [5], [8]. Conversely, the frequency of normal colonoscopies seemed greatly higher in our study comparing to a previous one that has a design that allowed comparison (14.2% vs 51%) [10]. This difference may, at least in part, be accounted for by varying definitions of what a normal colonoscopy is in the setting of chronic diarrhea.

The frequency of lymphocytic colitis was consistent with the one found in the prior study conducted in Peru [5]. Our findings consolidate the hypothesis that lymphocytic colitis is a frequent pathological diagnosis in the subpopulation of chronic diarrhea patients that undergo colonoscopy regardless of the socio-economic status which encompasses both dietary habits and levels of sanitation.

The complete abscence of the diagnosis of collagenous colitis in our series is congruous with Valle-Mansilla's et col findings (2 patients out of 110) [5]. This differs substantialy to studies conducted in the US and Europe, where the frequency of collagenous colitis was at least half the frequency of lymphocytic colitis [6], [7]. A more recent study in Egypt displays a similar trend [13]

Across international litterature the female to male ratio in lymphocytic colitis is around 2.4∶1. However in our study there is no substantial male or female predominance among patients with the finding of lymphocytic colitis (33 males, 25 females). This particularity could be accounted for by a well known limitation to access of women to non-reproductive healthcare in low and middle income countries such as Peru [14].

## Conclusions

In a 34 month study period 226 patients underwent colonoscopy as part of the workup for chronic diarrhea. The great majority of these patients had a macroscopically normal colon and terminal ileum. The most common colonic macroscopic lesions were ulcerated/erosive lesions. Having a macroscopically abnormal colon was associated with the pathological findings of granulomatous colitis as well as ulcerative colitis. The most common colonic mucosal histopathological finding was lymphocytic colitis which had an unusually high frequency compared to previous international reports.
